# Identification of soil bacteria capable of utilizing a corn ethanol fermentation byproduct

**DOI:** 10.1371/journal.pone.0212685

**Published:** 2019-03-08

**Authors:** Holly Packard, Zachary W. Taylor, Stephanie L. Williams, Pedro Ivo Guimarães, Jackson Toth, Roderick V. Jensen, Ryan S. Senger, David D. Kuhn, Ann M. Stevens

**Affiliations:** 1 Department of Biological Sciences, Virginia Tech, Blacksburg, VA, United States of America; 2 Department of Biological Systems Engineering, Virginia Tech, Blacksburg, VA, United States of America; 3 Department of Chemical Engineering, Virginia Tech, Blacksburg, VA, United States of America; 4 Department of Food Science and Technology, Virginia Tech, Blacksburg, VA, United States of America; National Centre For Cell Science, INDIA

## Abstract

A commercial corn ethanol production byproduct (syrup) was used as a bacterial growth medium with the long-term aim to repurpose the resulting microbial biomass as a protein supplement in aquaculture feeds. Anaerobic batch reactors were used to enrich for soil bacteria metabolizing the syrup as the sole nutrient source over an eight-day period with the goal of obtaining pure cultures of facultative organisms from the reactors. Amplification of the V4 variable region of the 16S rRNA gene was performed using barcoded primers to track the succession of microbes enriched for during growth on the syrup. The resulting PCR products were sequenced using Illumina MiSeq protocols, analyzed via the program QIIME, and the alpha-diversity was calculated. Seven bacterial families were the most prevalent in the bioreactor community after eight days of enrichment: *Clostridiaceae*, *Alicyclobacillaceae*, *Ruminococcaceae*, *Burkholderiaceae*, *Bacillaceae*, *Veillonellaceae*, and *Enterobacteriaceae*. Pure culture isolates obtained from the reactors, and additional laboratory stock strains, capable of facultative growth, were grown aerobically in microtiter plates with the syrup substrate to monitor growth yield. Reactor isolates of interest were identified at a species level using the full 16S rRNA gene and other biomarkers. *Bacillus* species, commonly used as probiotics in aquaculture, showed the highest biomass yield of the monocultures examined. Binary combinations of monocultures yielded no apparent synergism between organisms, suggesting competition for nutrients instead of cooperative metabolite conversion.

## Introduction

Commercial-level ethanol production is a global industry that yielded roughly 27 billion gallons in 2017, around 16 billions of which were processed in the United States [1]. Production of ethanol uses a starch-based biomass, with corn being the predominant source in U.S. production. Ethanol production commonly starts with milling of the corn, followed by cooking, liquification, and saccharification to allow yeast fermentation of the product, which is then distilled to separate ethanol from the stillage byproducts. The stillage is then centrifuged to separate the solid (wet distillers’ grains) from liquid (thin stillage) portions [2]. The thin stillage is concentrated through high temperature evaporation into condensed corn distillers solubles (CCDS), commonly termed syrup. This syrup is often used as an animal feed supplement when combined and dried with the wet distillers’ grains [2, 3, 4]. Since the syrup contains organic carbon sources [5], microbes have also been used to convert it into other desirable products, such as production of the complex polysaccharide pullulan by fungi (e.g. *Aureobasidium* sp.) [6, 7]. However, the syrup remains an underutilized component of the ethanol production process. In this study, the syrup was used as a nutrient-rich medium for microbial biomass development, which, if successful, would improve the profitability of ethanol production by developing a possible new protein source for aquaculture feeds.

Large-scale cultivation of microbial biomass has been used in a variety of industrial practices, including production of agricultural probiotics [8], carotenoid production [9], human and agricultural food production [10, 11, 12], and wastewater treatment [10, 13, 14, 15]. The aquaculture industry has a particular interest in culturing protein-rich bacterial biomass on wastewater/byproducts to replace fishmeal in aquaculture feeds. Fishmeal has traditionally served as the major protein source for many cultured species of fish and shellfish due to its high palatability and digestibility and its well-balanced essential amino acid profile [16]. However, fishmeal demand has increased along with the price (under $650/metric ton in 2003 to over $1,500/metric ton in 2018 [17]), which is making alternative protein sources more lucrative to the aquaculture industry. Culturing microbial biomass on wastewater/byproducts has the added benefit of making use of these otherwise no- to low-value waste streams. It has been previously demonstrated that microbial biomass can be cultured while treating wastewater from fish farms and confectionary manufacturing plants and this biomass can be successfully used to replace fishmeal in feeds for shrimp grown in aquaculture [18, 19]. The goal of this study was to investigate the capacity for microbes to grow on a corn ethanol fermentation syrup substrate so that they might also be used as a protein supplement in aquaculture feeds. A bioreactor-grown soil enrichment community capable of metabolizing the syrup was first established and its community profile was characterized at a molecular level using 16S rRNA sequences. Then, defined monocultures were tested for their growth yields, and binary culture combinations were examined for possible synergistic effects within the community. *Bacillus* species, although not the dominant organism in the bioreactor, were the most productive pure culture isolates. These findings lay the groundwork for future application of the bacterial biomass in aquaculture.

## Materials and methods

### The syrup growth substrate

Syrup, a byproduct of ethanol production, was obtained for used as a growth substrate for bacteria. To examine how robust the bacterial growth would be across different lots of syrup, three separate batches were provided from three different Flint Hills Resources (FHR) ethanol production facilities with similar design and function. All three facilities are an ICM design for 100 million gallons per year plants (ICM, Inc., KS, USA). The syrups were removed at the same point in the ethanol production process, after oil separation and evaporation through the application of centrifugation and high temperature (~85°C), respectively. Although three different commercial yeast strains were used, Bio-Ferm XR, TransFerm Yield+ (Lallemand Biofuels and Distilled Spirits, GA, USA) and Ethanol Red (Fermentis, France), the CCDS produced were all similar in content with ~30–40% solids and ~60–70% water. The CCDS, known as syrup, were cooled prior to shipping overnight to Virginia Tech. Upon receipt, an aliquot of each syrup was streaked on to a rich medium, trypticase-soy agar (TSA; 17 g L^-1^ pancreatic digest of casein, 5 g L^-1^ sodium chloride, 3 g L^-1^ papaic digest of soybean, 2.5 g L^-1^ dipotassium phosphate, 2.5 g L^-1^ dextrose), to ensure that there were no microbial contaminants present prior to further analysis. Using aseptic technique, the contaminant-free lots of syrup were individually diluted 1:1 (volume/volume) with milliQ dH_2_O. Bottles (500 mL) of the diluted syrup were centrifuged (Avanti J-26 XP centrifuge with JA-10 rotor, Beckman Coulter, CA, USA) for 30 min at 4,000 rpm and 4°C to remove insoluble solids. The syrup was then filtered through autoclaved Sofwipe cheesecloth (American Fiber & Furnishing, Inc., MA, USA) to remove additional remaining solids from the syrup and the processed liquid syrup was stored at 4°C for short-term use or -20°C for longer-term storage in sterile containers. This processed syrup had high water activity as it contained ~12–16% solids in ~84–88% water with the dominant solids/solutes comprised of ~27–34% glycerol, ~16–20% dextrin (DP4), ~9–11% maltose (DP2) with lesser amounts of maltotriose (DP3), glucose (DP1), and lactic acid detected across all three processed syrups used for further studies. Syrup 2 was used for all studies, while syrups 1 and 3 were examined in monoculture studies to test for organism robustness.

### Temporal community profiling of bacterial enrichment in anaerobic reactors

Processed syrup 2 was added to 1 L digestor reactors at a 1:16 (volume/volume) dilution with milliQ dH_2_O. A soil sample was obtained, 13 inches below the surface, from a site adjacent to a local cornfield with likely exposure to residuals from corn plants (latitude: 37.211668; longitude: -80.436833). Aliquots of the soil sample were added to reactors at a 1:10 mass to volume ratio with the diluted and processed syrup to enrich the microbial community for organisms capable of using the syrup as their sole nutrient source. Nitrogen gas was sparged through the reactors for 5 min to create an anaerobic environment that would select against strict aerobes. Anaerobic digestion was performed at 37°C over a four-day initial enrichment period without any adjustment to the pH. On the fourth day, the liquid top layer was removed from the denser solid and particulate materials (i.e. soil) and replaced with fresh processed syrup, and this second enrichment continued for another four days. In total, the microbial community was enriched over an eight-day period. Samples (10 mL) were collected via a sampling syringe and deposited into sterile conical tubes aerobically on day 0, day 1, and day 4 from the first portion of the enrichment cycle. After the addition of more syrup, samples were obtained on day 5 and day 8 from the second portion of the enrichment cycle, totaling five samples each from three separate reactors. DNA was extracted from these samples using the Qiagen DNeasy Powersoil Kit (Qiagen, MD, USA) per the manufacturer’s protocol. PCR was performed in triplicate using 0.2 μM barcoded primers for the V4 variable region of the 16S rRNA gene as previously described (S1 Table) [20], as well as 1X 5Prime HotMasterMix (Quantabio, MA, USA), and chromosomal DNA template from each enrichment time point. Thermocycler conditions were set for an initial denaturation at 94°C for 2 min, followed by 35 cycles of denaturation at 94°C for 5 min, annealing at 50°C for 1 min, and elongation at 68°C for 1.5 min, with a final extension cycle at 68°C for 10 min before holding at 4°C. A negative control PCR for each primer set was performed without template. The triplicate samples were combined, visualized on a 1% agarose gel, and purified using the PCR Purification Kit (Qiagen). The concentration of each sample was measured using Qubit at the Virginia Tech Biocomplexity Institute (VTBI), and samples pooled for 250 nt paired-end read Illumina MiSeq (VTBI) [21].

### MiSeq data analysis

The Illumina sequencing data generated for the forward sequences was 15,377,857 total sequences, with 4,455,215 of those reads unassigned to a specific sample due to insufficient barcode sequence quality. Sequencing data was provided demultiplexed into the 15 original samples (five time points, three replicate digestors each) and analyzed with the program Quantitative Insights Into Microbial Ecology (QIIME, v. 1.9.1). Individual samples ranged between 317,933–816,771 sequences. Each sample was quality filtered to remove sequences shorter than 200 nucleotides, sequences with a phred score less than 20, and those with more than six ambiguous bases. After quality filtering, the new sequence range was 286,912–714,010, so all samples were rarefied to 286,900 reads. These were then clustered via UCLUST into operational taxonomic units (OTUs) with a threshold of 97% similarity [22]. An OTU threshold minimum set at 0.001% of the total sequences was implemented, resulting in the total sequence count for the OTU table to be 8,658,900 reads. OTU tables were generated based on these rarefied values and exported into a spreadsheet. A Shannon index was calculated to give the alpha diversity across all three replicate reactor samples for each time point, and the estimated number of families (ENF) was also calculated (Table 1) [23].

**Table 1:** Estimated number of families and alpha diversity of the soil enrichment community.

Sample Time Point ^a^	Estimated Number of Families^b^	Alpha Diversity ^b^
Day 0	22	3.1
Day 1	12	2.5
Day 4	5.2	1.7
Day 5	3.7	1.3
Day 8	8.0	2.1

^a^ Calculations made using data from three pooled PCR technical replicates from each of the three replicate reactors.

^b^ Shannon index used for alpha diversity calculations and ENF calculation.

### Pure culture strain isolation from enrichment cultures

At the end of an eight-day enrichment experiment, the solid materials from one anaerobic reactor were used to obtain 10 pure isolated colonies for follow-up monoculture studies. The anaerobic reactors were not run to achieve a steady state; instead, the short time frame of the enrichment was designed to select for the fastest growing organisms. In addition, sampling throughout the enrichment process by intention was not kept strictly anaerobic to select for facultative organisms. Fast growing, facultative organisms were preferable for possible downstream commercial applications. Therefore, subsequent organism isolation was performed in parallel using both anaerobic and aerobic conditions in batch culture in an attempt to obtain a diversity of organisms in pure culture. Part of the sample was transferred to a Coy anaerobic chamber (Coy Laboratory Products, MI, USA), where it was T-streaked onto TSA and grown anaerobically for 48 hr at 37°C. Colonies of various morphologies were then purified into separate stocks for further use and designated UAN (Unknown Anaerobe; Table 2). This isolation process was repeated aerobically, and purified stocks were designated UAE (Unknown Aerobe; Table 2).

**Table 2:** Microbes used in this study^a^.

Strain Code	Strain Identity	Source	Sequenced Gene Target
M1	*Bacillus subtilis*	ATCC 23857	Known–not sequenced
M2	*Bacillus licheniformis*	ATCC 9945A	Known–not sequenced
M3	*Bacillus subtilis*	ATCC 6051	Known–not sequenced
M5	*Enterococcus faecium*	ATCC 19434	Known–not sequenced
M6	*Enterococcus lactis*	ATCC 11454	Known–not sequenced
M7	*Saccharomyces cerevisiae*	Virginia Tech Teaching Labs	Known–not sequenced
M8	*Bifidobacterium lactis*	Commercial yogurt isolate	Known–not sequenced
M9	*Pantoea stewartii* subsp. *stewartii* DC283	[24]	Known–not sequenced
M11	*Pichia kudriavzevii*	Syrup contaminant	ITS2 region
UAE1	*Bacillus cereus*, *Bacillus mycoides*, *Bacillus subtilis*, *Bacillus thuringiensis*, *Bacillus weihenstephanensis*	Soil enrichment isolate	16S rRNA gene
UAE2	*Bacillus pumilus*, *Bacillus safensis*	Soil enrichment isolate	16S rRNA gene, *gyrB*, *rpoB*, *pyrE*
UAE3	*Bacillus cereus*, *Bacillus mycoides*, *Bacillus thuringiensis*, *Bacillus toyonensis*, *Bacillus weihenstephanensis*	Soil enrichment isolate	16S rRNA gene
UAE4	*Bacillus amyloliquefaciens*, *Bacillus subtilis*, *Bacillus velezensis*	Soil enrichment isolate	16S rRNA gene, *gyrB*, *rpoB*, *pyrE*
UAE5	*Bacillus pumilus*, *Bacillus safensis*	Soil enrichment isolate	16S rRNA gene, *gyrB*, *rpoB*, *pyrE*
UAE6	*Bacillus anthracis*, *Bacillus cereus*, *Bacillus subtilis*, *Bacillus thuringiensis*	Soil enrichment isolate	16S rRNA gene
UAE7	*Bacillus anthracis*, *Bacillus cereus*, *Bacillus toyonensis*, *Bacillus thuringiensis*	Soil enrichment isolate	16S rRNA gene
UAE8	*Bacillus anthracis*, *Bacillus cereus*, *Bacillus toyonensis*, *Bacillus thuringiensis*	Soil enrichment isolate	16S rRNA gene
UAE10	*Bacillus cereus*, *Bacillus mycoides*, *Bacillus pseudomycoides*	Soil enrichment isolate	16S rRNA gene
UAN4	*Bacillus anthracis*, *Bacillus cereus*, *Bacillus thuringiensis*	Soil enrichment isolate	16S rRNA gene

^a^Multiple species listed for organisms with 100% sequencing identity using the listed gene(s) as sequencing targets.

One additional monoculture, M11, was isolated aerobically from a contaminated batch of syrup received from FHR that was not used as a growth substrate. The source of this contaminant is unknown.

### Monoculture and binary-combination growth assays of microbial strains

Strains of interest were grown overnight at 37°C with shaking at 250 rpm in trypticase-soy broth (TSB; Table 2) and subcultured into fresh medium to a 1:100 dilution, followed by growth for four hr. These actively growing cultures were then each diluted in phosphate buffered saline (PBS; 137 mM NaCl, 2.7 mM KCl, 10 mM Na_2_HPO_4_ and 2 mM KH_2_PO_4_, pH 7.4) to an OD_600_ of 0.1. After dilution, 1.4 mL of the suspended cells were pelleted via centrifugation (Eppendorf 5424R centrifuge with FA-45-24-11 rotor, Germany) for 5 min at 5,000 rpm, washed with 0.5 mL PBS, and recentrifuged. The pellet was resuspended in 1.4 mL PBS then diluted 1:10 with PBS to a final OD_600_ of 0.01.

Each of the three processed syrups (batches 1–3) from the three corn ethanol production facilities was separately diluted 1:4 (volume/volume) with milliQ dH_2_O, and 100 μL added to wells in the Nunclon Delta Surface 96-well plate (Thermo Scientific, MA, USA). No pH adjustment was made to the growth medium. For the monoculture growth assays, 100 μL culture in PBS (OD_600_ of 0.01) was added to the three syrups (final processed syrup dilution of 1:8; final OD_600_ at 0.005) in triplicate. Triplicate wells of each syrup with 100 μL PBS were used as negative controls to ensure that there was no microbial contamination from the syrup, and cultures with 100 μL TSB were used as positive viability controls. Microtiter plates were incubated at 37°C in standing conditions for four days. Absorbance readings (600 nm) were taken using the SpectraMax M5 spectrophotometer (Molecular Devices, CA, USA) each day, including an initial reading. For the binary culture assays, cultures were grown and washed separately, then 50 μL of each were added to the appropriate well (S2 Table). Binary culture assays only used syrup 2.

### Strain identification

Unknown isolates (Table 2) were grown overnight at 37°C in TSB. Microscopy and a Gram stain were performed on each of the unknown monocultures. DNA from Gram-positive bacterial monocultures was extracted using the DNeasy Blood and Tissue Kit (Qiagen) per the manufacturer’s recommended protocol. For the M11 yeast strain, cells grown overnight at 37°C on TSA were added via toothpick to 300 μL of NTES (0.5 M NaCl, 0.2 Tris HCl, pH 7.5, 0.01 M Na_3_ EDTA, 1% SDS). Glass beads (300–650 μL; G-8772, acid-washed, Sigma-Aldrich, MO, USA) and 300 μL 1:1 phenol:chloroform were added and vortexed for 7 min at 4°C. After centrifugation at 12,000 rpm (Eppendorf 5417R centrifuge with F45-30-11 rotor) for 10 min at 4°C, the aqueous phase was transferred to 800 μL cold ethanol and stored at -20°C overnight. Then, samples were centrifuged for 10 min at 4°C, washed with 70% ethanol, recentrifuged, then dried and dissolved in 30 μL dH_2_O [25]. PCR was performed using 0.2 μM of universal primers, 515F and 806R, designed for the 16S rRNA gene for strain identification, or the internal transcribed spacer (ITS2) region for the yeast identification (S1 Table), as well as 1X One*Taq* Quick-Load Master Mix, and chromosomal DNA template of each unknown microbe species. Thermocycler conditions were per enzyme protocol (New England Biolabs, MA, USA). Initial denaturation was at 94°C for 30 sec, followed by 30 cycles of denaturation at 94°C for 30 sec, annealing at 45°C for 1 min, and extension at 68°C for 1.5 min. Final extension was at 68°C for 5 min. More specific identification for select bacterial samples was performed using primers for housekeeping genes *gyrB*, *pyrE*, and *rpoB* [26]. PCR was performed as above with each appropriate primer (S1 Table), and thermocycler conditions were identical except for a shortened extension time at 68°C for 45 sec with 30 cycles. Products of all reactions were visualized on a 1% agarose gel. These were extracted via a Gel Extraction Kit (Qiagen) and sequenced (VTBI). The Basic Local Alignment Sequencing Tool (BLAST; NCBI) was used to determine the sequence identities via a nucleotide Megablast search within the nucleotide collection (nt/nr) and 16S rRNA sequences databases. Those with the highest Max Score (highest query and identity percentages, and E-value closest to 0.0) were used as the identity of the unknown organisms (Table 2).

### Accession numbers

Paired-end sequencing reads from the 15 samples of enriched bacterial communities (i.e. five time points with combined triplicate samples from three separate reactors) with syrup were deposited into the NCBI Sequence Read Archive (SRA) with accession number SRP144322.

## Results

### Community profiling illustrates succession of enriched bacterial families

**Fig 1:**
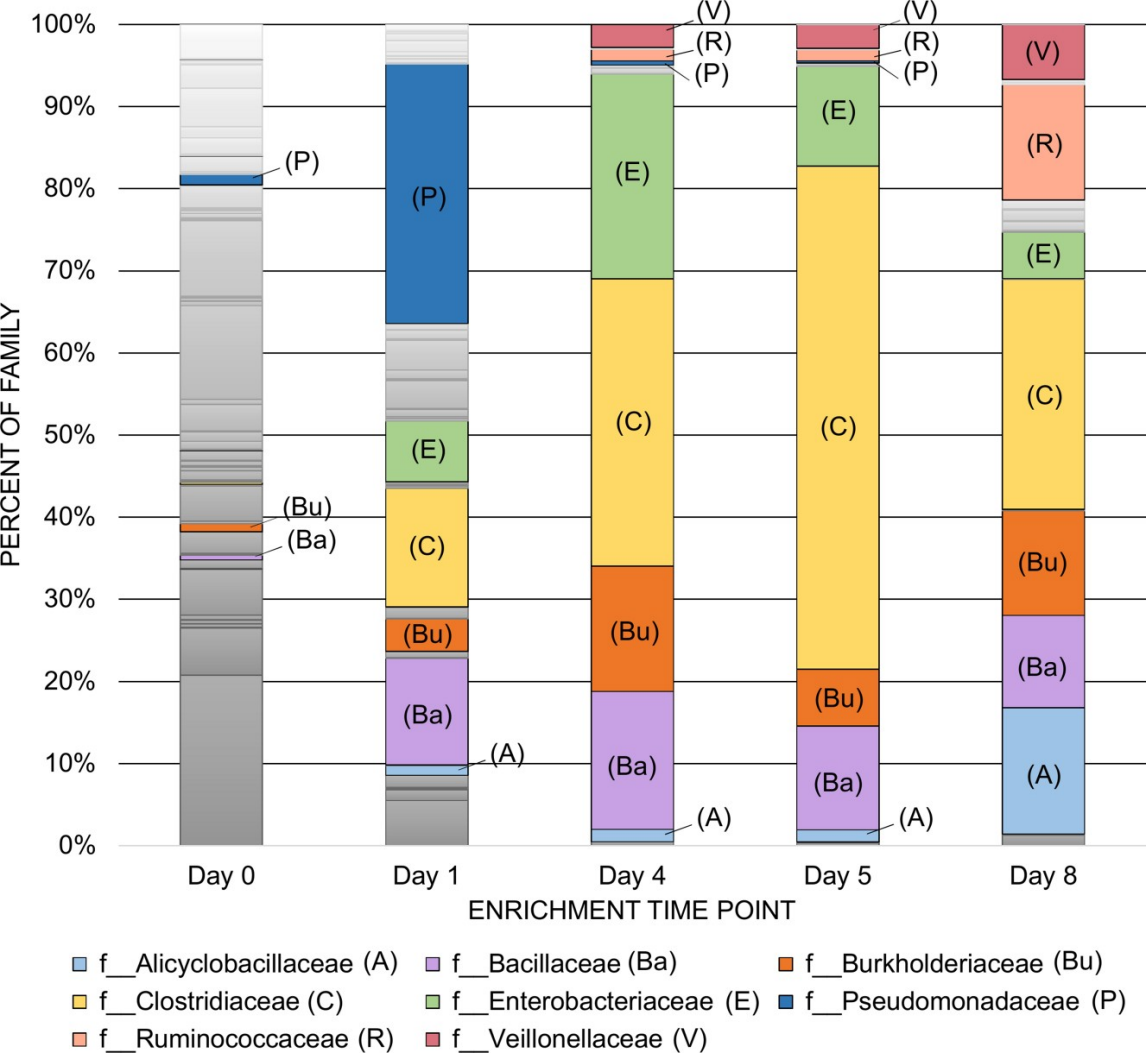
Bacterial community profile across anaerobic syrup enrichment cycles. Reads were averaged from the PCR pools for each of the three replicate reactors across five time points over the eight-day enrichment. Dominant families listed throughout the enrichment are indicated with the following labels: A = *Alicyclobacillaceae*, Ba = *Bacillaceae*, Bu = *Burkholderiaceae*, C = *Clostridiaceae*, E = *Enterobacteriaceae*, P = *Pseudomonadaceae*, R = *Ruminococcaceae*, and V = *Veillonellaceae*.

Anaerobic digestor reactors were used to enrich for soil bacteria capable of using syrup produced as a byproduct of corn ethanol production for their sole growth substrate with the goal of obtaining pure cultures of facultative organisms from the reactors. Temporal sampling for three separate reactors was performed to obtain five samples across an eight-day digestion enrichment to determine the bacterial community composition via 16S rRNA gene sequencing so that this profile could later be compared to the list of organisms isolated in culture. This also provided insights into the different families of organisms capable of using the syrup as a growth medium. For each of the three reactors utilized, solid materials from the bottom of the reactor were processed to purify DNA and then PCR was performed in triplicate. The PCR products were combined to produce the five temporal samples per reactor (i.e. 15 samples in total). At each time point the data from samples for the three reactors was averaged for determining the percent of dominant families present across all of the reactors (Fig 1). Additionally, the data was also used for the calculation of the alpha diversity and ENF. Both of these calculations showed an overall decrease in diversity as time progressed, with a slight increase toward the end of the enrichment period examined (Table 1). The system was intentionally not run to a steady state as the most rapidly growing organisms were desirable for downstream applications. The dominant families fluctuated over the eight-day enrichment, beginning with a very large increase in the *Pseudomonadaceae* family on the first day of enrichment (Fig 1). Subsequently, by the fourth day, organisms belonging to the *Clostridiaceae* family dominated the reactors at an average level of 35%, with *Enterobacteriaceae*, *Bacillaceae* and *Burkholderiaceae* following at 25%, 17%, and 15% respectively. After the removal of the liquid top layer and addition of fresh syrup, *Clostridiaceae* became even more dominant with 61% of reads belonging to that family on day five. On the final eighth day, 93.7% of the organisms present in the community belonged to the *Clostridiaceae* (28.0%), *Alicyclobacillaceae* (15.4%), *Ruminococcaceae* (14.0%), *Burkholderiaceae* (12.7%), *Bacillaceae* (11.3%), *Veillonellaceae* (6.7%), and *Enterobacteriaceae* (5.7%) families, and were more evenly present. The total number of families found across all three reactors ranged from an initial 66–81 on day zero to only 19–24 after eight days.

### Monoculture growth revealed differences in growth yield between strains

Solid material from the final day of bioreactor enrichment (day eight) was plated onto TSA under both aerobic and anaerobic conditions. A total of 10 pure-culture isolates were randomly selected from these agar plates to test for growth yield with the syrup as the sole nutrient source. An additional eight known stock cultures (designated M for pure monoculture), seven of which were lab stocks and the last obtained from a commercial yogurt product were also utilized (Table 2). Another organism, M11, was separately isolated from a contaminated batch of syrup unusable as a growth substrate. All newly isolated enrichment strains were identified via microscopy and a Gram stain as Gram-positive bacteria except M11, which was putatively identified as a yeast strain from the cell size and morphology. The aerobic (UAE) and anaerobic (UAN) isolates obtained from the reactors that maintained viability under facultative growth conditions, desired for future work, were monitored for their growth yield.

Monoculture assays were performed on three lots of syrup from different production facilities to examine the robustness of growth across similar, but not identical growth substrates. Growth occurred on all three syrups, but was not equivalent across the different syrups or the different organisms (Table 3 and S1 Fig). The most rapid phase of growth was between day 0 and day 1, with the majority of strains achieving their maximum yield by day 1 (S1 Fig). For the monocultures examined, the day 1 growth levels were highest, on average, for pure culture isolates M1-3 and M11 and enrichment culture isolates UAE2, UAE4, UAE5 and UAN4 across the three syrups (Table 3, gray highlights). Thus these strains exhibited the desire characteristics of rapid growth and robustness across a variety of similar substrates.

**Table 3:** Growth yields of monocultures on syrup from three production facilities^a^.

Monoculture	Syrup 1	Syrup 2	Syrup 3	Syrup Average
M1	0.28 ± 0.12	0.41 ± 0.23	0.58 ± 0.01	0.43 ± 0.12
M2	0.09 ± 0.10	0.41 ± 0.07	0.47 ± 0.04	0.32 ± 0.17
M3	0.40 ± 0.21	0.47 ± 0.27	0.57 ± 0.02	0.48 ± 0.07
M5	0.04 ± 0.01	0.15 ± 0.01	0.18 ± 0.03	0.13 ± 0.06
M6	0.10 ± 0.01	0.26 ± 0.01	0.20 ± 0.02	0.19 ± 0.07
M7	0.08 ± 0.01	0.11 ± 0.01	0.20 ± 0.04	0.13 ± 0.05
M8	0.15 ± 0.00	0.28 ± 0.01	0.26 ± 0.04	0.23 ± 0.06
M9	0.02 ± 0.01	0.06 ± 0.02	0.05 ± 0.01	0.04 ± 0.02
M11	0.56 ± 0.02	0.75 ± 0.07	0.56 ± 0.05	0.62 ± 0.09
UAE1	0.02 ± 0.01	0.38 ± 0.24	0.18 ± 0.18	0.20 ± 0.15
UAE2	0.10 ± 0.06	0.29 ± 0.02	0.52 ± 0.06	0.30 ± 0.17
UAE3	0.03 ± 0.02	0.61 ± 0.18	0.12 ± 0.04	0.26 ± 0.25
UAE4	0.35 ± 0.02	0.68 ± 0.05	0.53 ± 0.02	0.52 ± 0.13
UAE5	0.11 ± 0.06	0.49 ± 0.08	0.53 ± 0.10	0.38 ± 0.19
UAE6	0.03 ± 0.02	0.35 ± 0.03	0.23 ± 0.03	0.20 ± 0.13
UAE7	0.01 ± 0.01	0.29 ± 0.08	0.24 ± 0.09	0.18 ± 0.13
UAE8	0.02 ± 0.00	0.26 ± 0.07	0.16 ± 0.04	0.15 ± 0.10
UAE10	0.01 ± 0.01	0.35 ± 0.01	0.07 ± 0.06	0.14 ± 0.15
UAN4	0.01 ± 0.01	0.76 ± 0.05	0.13 ± 0.03	0.30 ± 0.33

^a^M = monoculture from laboratory isolates. UAE = unknown aerobically plated environmental isolate. UAN = unknown anaerobically plated environmental isolate. Optical density data (OD_600_) readings from day 1 of growth averaged from three replicate growth assay plates, with data from each plate an average of three replicate wells. Background correction performed by subtracting averages of syrup with PBS wells from each plate. Standard deviations calculated for each individual syrup experiment across the independent replicate plates, and across the independent syrup experiments. Gray boxes denote the organisms with the highest average growth across all syrups.

### Binary culture combinations showed no synergism

To explore whether two monocultures together would result in an increased maximum growth yield, binary combination growth assays were performed using select monoculture strains with syrup 2 as the medium, since it was also the substrate used in the bioreactors (S2 Table). Growth patterns in the binary assays showed a predominant number of combinations (30 out of 34) to have very similar growth trends with at least one of the original monoculture growth patterns, suggesting no synergistic effects between those organisms (S2 Fig). Two combinations, M2 with M6 and M2 with M7, resulted in a yield that was intermediate between the two monoculture growth patterns, as each organism alone had a very different yield (S2 Fig). M11 with UAE2 and M11 with UAE5 did show slight increases in combined yield, but these were not considered to be important differences (S2 Fig). These results suggest that for the culture combinations examined, neither organism present generated a product(s) that noticeably aided in the growth of the other organism.

### Pure-culture isolates from reactors were *Bacillus* species

Industrial application of cultured biomass for animal consumption requires knowledge of the exact composition and growth capabilities of the organisms present to ensure safety. To determine the identification of the UAE and UAN enrichment isolates used in the microtiter growth assays, and to correlate this information to the community profiles found in the bioreactors, the 16S rRNA gene from each enrichment monoculture isolate was subject to sequencing. Sequencing results revealed that all of them belonged to the *Bacillus* genus (Table 2). The conserved nature of the 16S rRNA gene within the *Bacillus* genus hindered identification of each isolate at the species level. Because the biomass was proposed to be used for animal consumption followed by human consumption, any sequencing result that suggested an organism might be a potential pathogen, such as *Bacillus anthracis* or *Bacillus cereus*, led to the organism being removed from further consideration. Therefore, additional characterization at the species level was performed on UAE2, UAE4, and UAE5 using genes *gyrB*, *pyrE*, and *rpoB* for a more specific species determination. UAE2 and UAE5 were both found to be either *Bacillus pumilus* or *Bacillus safensis*, while UAE4 was found to be either *Bacillus amyloliquefaciens*, *Bacillus subtilis* or *Bacillus velezensis* (Table 2). Thus, the UAE2, UAE4 and UAE5 isolates are considered to be candidates for future work to develop them into safe direct fed protein supplements for aquaculture feed, in addition to the pure culture isolates M1, M2 and M3.

## Discussion

The purpose of this study was to identify pure culture isolates obtained from laboratory stocks and a soil enrichment community that could grow using a byproduct of ethanol fermentation production as their sole nutrient source. At the final time point (day eight) of the anaerobic digestion with the syrup and soil (Fig 1), there were seven dominant organism families that were enriched including the *Bacillaceae* family that proved to be the group of organisms of greatest interest. The other six families enriched in the bioreactor were *Clostridiaceae*, *Alicyclobacillaceae*, *Ruminococcaceae*, *Burkholderiaceae*, *Veillonellaceae*, and *Enterobacteriaceae*. However, of those enriched, only four, the *Bacillaceae*, *Burkholderiaceae*, *Alicyclobacillaceae*, and *Enterobacteriaceae* families, have members that are facultative anaerobes. Our procedures were designed to select against organisms that were obligate aerobes or anaerobes. Facultative growth is an ideal trait that enables handling of organisms under aerobic conditions, while allowing fermentation in large-scale industrial vats.

The monoculture assays revealed *Bacillus* species as the organisms capable of the highest levels of growth with the syrup substrate. While the most productive monocultures exclusively belonged to the *Bacillaceae* family, the enrichment study revealed a more diverse community of seven dominant families capable of growth on the syrup. In fact the *Bacillaceae* family represented just 11.3% of the total community. Antagonism and/or competition were probable contributing factors within the reactors limiting the growth of the *Bacillaceae*. However, the *Bacillaceae* were the most successful group of organisms at adapting to the different selections we applied with regard to oxygen availability (i.e. anaerobic and aerobic growth) and medium choice (TSA and syrup). Since variability in syrup composition and nutritional content is known [27], a highly desirable trait is the capacity for robust growth across different batches. The *Bacillaceae* examined appear to have this desired characteristic.

Interestingly, numerous studies have shown *Bacillus* sp. to be able to utilize multiple carbon sources, and they are capable of catabolite repression when grown in the presence of more than one source [28,29]. More recently, interest in *Bacillus* sp. metabolism has increased regarding carbon sources that are industrially relevant, including the anhydrosugar levoglucosan from burning biomass or hemicellulose from plant residues [30–32]. Since the process used by the FHR production facilities does not entail very high temperatures, with all steps occurring at less than 87.8°C (190°F) it would be unlikely that anhydrosugars would be formed. Instead, glycerol, dextrin (DP4), and maltose (DP2) were the most abundant carbohydrates in the solid fraction of the FHR syrups.

Thirty-four binary combinations of the microbes were grown on the syrup to see if the initial syrup substrate might be interconverted into metabolites better supporting growth of the mixed community. The binary growth assays revealed no apparent synergistic growth effects, as none of the combinations tested grew any better than did just one of the individual organisms (S2 Fig). This indicates that there are no beneficial metabolites produced for the paired organisms to use. Despite this, synergism could be possible between the top *Bacillus* species isolates and other organisms that were present in the initial bioreactor community.

The discovery that *Bacillus* species utilize the syrup as their sole nutrient source has the potential for future applications. For example, bacteria in the *Bacillus* genus have been used as a supplement with aquaculture feed in industrial practices, specifically for probiotic benefit, stimulating fish immune system, and even improving water quality [8]. In addition, their ability to form highly stable dormant spores makes long-term transport and storage possible [33, 34]. By dry weight, *Bacillus* cells are roughly 50% protein, thus they could be used as a supplement instead of expensive fishmeal in the feed of aquaculture-grown animals. This study identified some promising *Bacillus* isolates that would be considered safe for animal consumption as a substitute for fishmeal.

In 2016, about 20 million tons of global fish production went toward fishmeal or fish oil use, almost 12% of total fish production worldwide [16]. A rise in demand due to increased aquaculture practices combined with the irregular supply of fishmeal due to overfished marine environments has resulted in a heightened cost of fishmeal [16, 17]. These trends suggest a need for additional and alternative nutritional material for a permanent aquaculture feed supplement. Therefore, the use of the syrup substrate as a means to cultivate microbes, such as *Bacillus* species, would provide enhanced economic and sustainability benefits not only to the process of ethanol production, but also to commercial aquaculture.

## Supporting information

S1 Dataset (XLSX)

S1 Fig Growth rates and yields of laboratory strains and enrichment isolates grown in monoculture on syrup from three production facilities. M = monoculture from laboratory isolates. UAE = unknown aerobically plated environmental isolate. UAN = unknown anaerobically plated environmental isolate. Data averaged from three replicate growth assay plates, with data from each plate an average of three replicate wells across four days. Error bars were estimated using the sample standard error of the log absorbance across three independent replicate plates.
(TIFF)

S2 Fig Growth of binary combinations of laboratory strains and environmental isolates. Graphs depicting example trends for intermediate combination growth (A), combination growth aligning with monoculture growth (B), or slight increase in combination growth compared to monocultures (C). Syrup 2 was used for all experiments. Data averaged from three replicate plates, with data from each plate an average of three replicate wells. Error bars were estimated with the sample standard error of the log absorbance across three independent replicate plates.
(TIFF)

S1 Table Primers used in this study^a,b^. ^a^Barcodes for the 16S rRNA gene V4 region forward primers are indicated by the underlined sequences. Remaining sequence includes adapter, primer pad, linker, and actual primer sequence, which are all identical for the barcoded primers. Reactor samples are designated by replicate (R) and day of enrichment (D). An example is R1D4, a sample from the first reactor replicate, taken on the fourth day of enrichment. ^b^EMP = Earth Microbiome Project (Gilbert *et al*., 2014), ITS2 = internal transcribed spacer region 2
(DOCX)

S2 Table Binary growth combinations performed with laboratory strains and environmental isolates^a^. ^a^Combinations performed are designated with “X”
(DOCX)
