# Supplementary figures and images for: Identification of soil bacteria capable of utilizing a corn ethanol fermentation byproduct
Source: PLoS One. 2019 Mar 8;14(3):e0212685. doi: 10.1371/journal.pone.0212685 (PMC6407766; doi:10.1371/journal.pone.0212685)

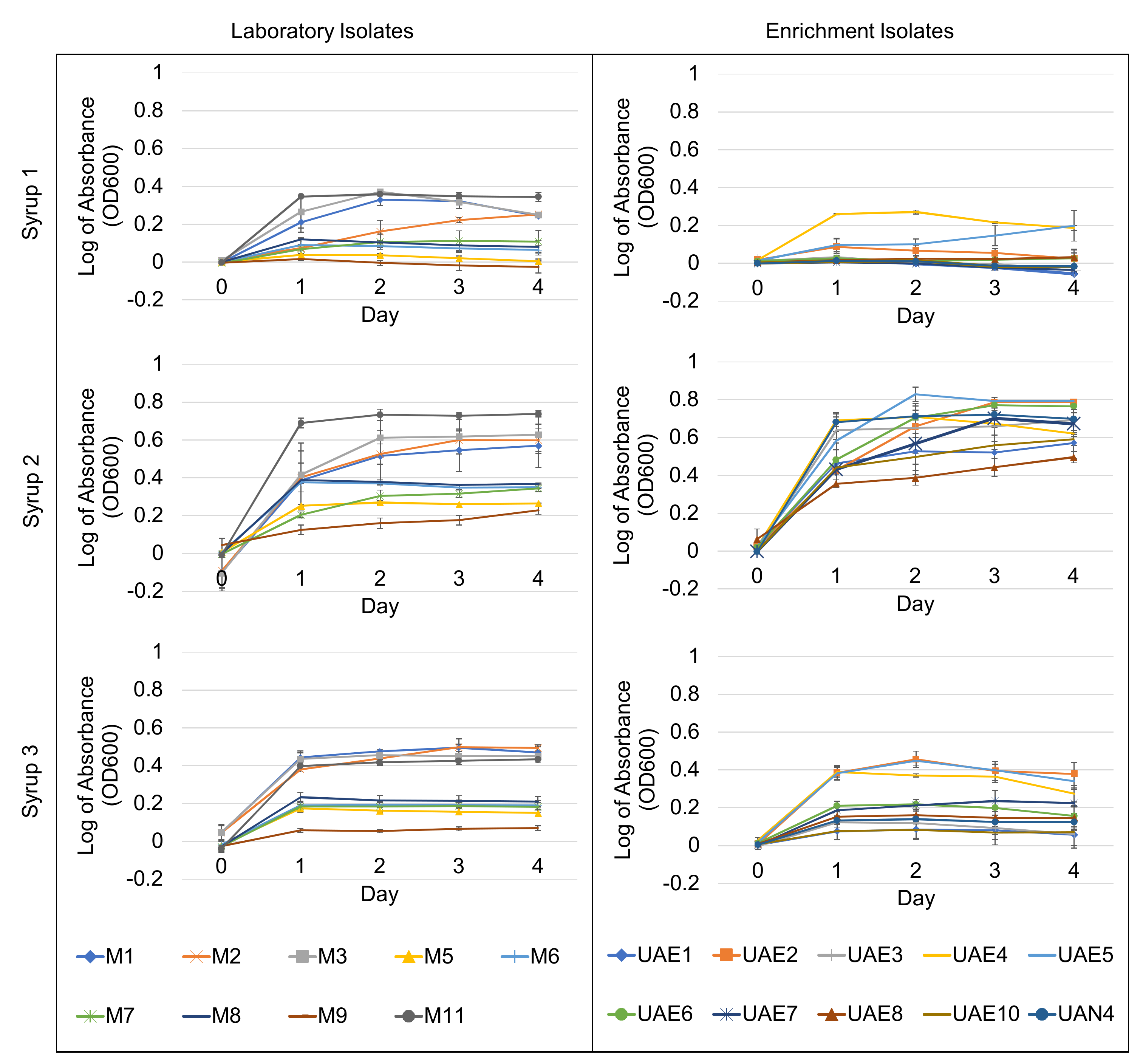

Supplement: S1 Fig — M = monoculture from laboratory isolates. UAE = unknown aerobically plated environmental isolate. UAN = unknown anaerobically plated environmental isolate. Data averaged from three replicate growth assay plates, with data from each plate an average of three replicate wells across four days. Error bars were estimated using the sample standard error of the log absorbance across three independent replicate plates. (TIFF) [file pone.0212685.s002.tiff]

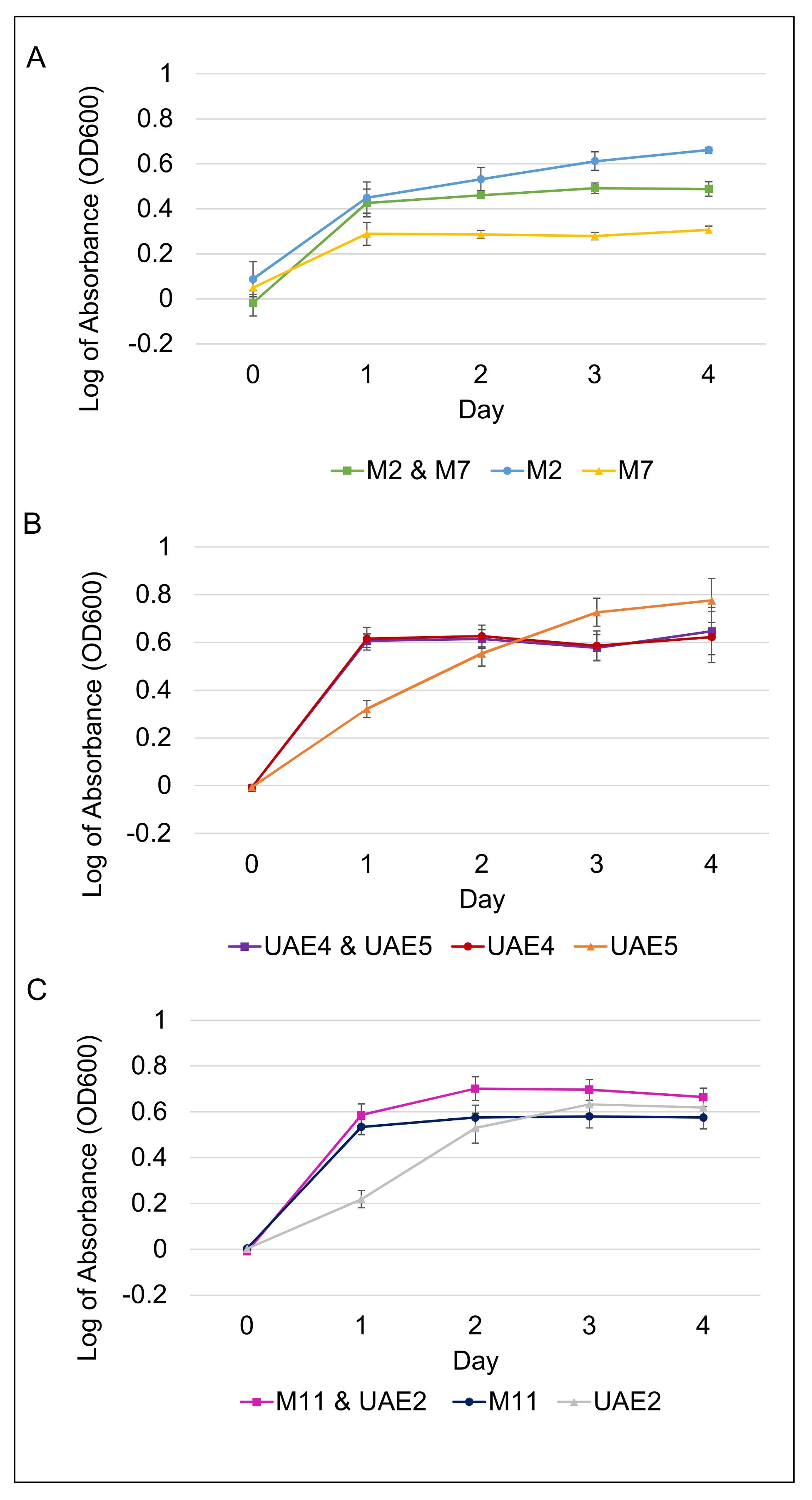

Supplement: S2 Fig — Graphs depicting example trends for intermediate combination growth (A), combination growth aligning with monoculture growth (B), or slight increase in combination growth compared to monocultures (C). Syrup 2 was used for all experiments. Data averaged from three replicate plates, with data from each plate an average of three replicate wells. Error bars were estimated with the sample standard error of the log absorbance across three independent replicate plates. (TIFF) [file pone.0212685.s003.tiff]
