# Supplementary material for: Identification of soil bacteria capable of utilizing a corn ethanol fermentation byproduct
Source: PLoS One. 2019 Mar 8;14(3):e0212685. doi: 10.1371/journal.pone.0212685 (PMC6407766; doi:10.1371/journal.pone.0212685)
Supplement: S1 Table — aBarcodes for the 16S rRNA gene V4 region forward primers are indicated by the underlined sequences. Remaining sequence includes adapter, primer pad, linker, and actual primer sequence, which are all identical for the barcoded primers. Reactor samples are designated by replicate (R) and day of enrichment (D). An example is R1D4, a sample from the first reactor replicate, taken on the fourth day of enrichment. bEMP = Earth Microbiome Project (Gilbert et al., 2014), ITS2 = internal transcribed spacer region 2 (DOCX) [file pone.0212685.s004.docx]

S1 Table. Primers used in this study^a,b^

| Amplification Target and Purpose | Sequence | Source |
| --- | --- | --- |
| 16S rRNA gene coding sequence (27F-1492R) | F: AGA GTT TGA TCM TGG CTC AG  R: ACC TTG TTA CGA CTT | Weisburg *et al*., 1991 |
| ITS2 region | F: AGGAGAAGTCGTAACAAGGT  R: TCCTCCGCTTATTGATATGC | White *et al*., 1990 |
| *gyrB* partial coding sequence | F: TAC ATC GGA TCA ACT AAC AGC  R: CGT ATC AGT GAT CGT TCT CG | This study |
| *pyrE* partial coding sequence | F: TTT ACG CCC GAA TGA GCC  R: TTG ACT CGG GAT TCT GTT TCC | This study |
| *rpoB* partial coding sequence | F: GTT GGC TTC ATG ACT TGG GA  R: ACG TTC CAT ACC TAA ACT TTG | Liu *et al*., 2013 |
| Illumina MiSeq sequencing | F: TAT GGT ATT TGT GTG YCA GCM GCC GCG GTA A  R: AGT CAG CCA GCC GGA CTA CNV GGG TWT CTA AT  Index: ATT GAT ACG GCG ACC ACC GAG ATC TAC ACG CT | EMP |
| 16S rRNA gene V4 region reverse primer (with adapter, primer pad, linker) | CAA GCA GAA GAC GGC ATA CGA GAT AGT CAG CCA GCC GGA CTA CNV GGG TWT CTA AT | EMP |
| 16S rRNA gene V4 region forward primer: R1D0 | AATGATACGGCGACCACCGAGATCTACACGCTAAGTCACACACATATGGTAATTGTGTGYCAGCMGCCGCGGTAA | EMP |
| 16S rRNA gene V4 region forward primer: R1D1 | AATGATACGGCGACCACCGAGATCTACACGCTTTACTTATCCGATATGGTAATTGTGTGYCAGCMGCCGCGGTAA | EMP |
| 16S rRNA gene V4 region forward primer: R1D4 | AATGATACGGCGACCACCGAGATCTACACGCTGTGTCGAGGGCATATGGTAATTGTGTGYCAGCMGCCGCGGTAA | EMP |
| 16S rRNA gene V4 region forward primer: R1D5 | AATGATACGGCGACCACCGAGATCTACACGCTACGGCGTTATGTTATGGTAATTGTGTGYCAGCMGCCGCGGTAA | EMP |
| 16S rRNA gene V4 region forward primer: R1D8 | AATGATACGGCGACCACCGAGATCTACACGCTAGTTGTAGTCCGTATGGTAATTGTGTGYCAGCMGCCGCGGTAA | EMP |
| 16S rRNA gene V4 region forward primer: R2D0 | AATGATACGGCGACCACCGAGATCTACACGCTGAAGTAGCGAGCTATGGTAATTGTGTGYCAGCMGCCGCGGTAA | EMP |
| 16S rRNA gene V4 region forward primer: R2D1 | AATGATACGGCGACCACCGAGATCTACACGCTATGGGACCTTCATATGGTAATTGTGTGYCAGCMGCCGCGGTAA | EMP |
| 16S rRNA gene V4 region forward primer: R2D4 | AATGATACGGCGACCACCGAGATCTACACGCTTTCCACACGTGGTATGGTAATTGTGTGYCAGCMGCCGCGGTAA | EMP |
| 16S rRNA gene V4 region forward primer: R2D5 | AATGATACGGCGACCACCGAGATCTACACGCTGAACCGTGCAGGTATGGTAATTGTGTGYCAGCMGCCGCGGTAA | EMP |
| 16S rRNA gene V4 region forward primer: R2D8 | AATGATACGGCGACCACCGAGATCTACACGCTAGGGACTTCAATTATGGTAATTGTGTGYCAGCMGCCGCGGTAA | EMP |
| 16S rRNA gene V4 region forward primer: R3D0 | AATGATACGGCGACCACCGAGATCTACACGCTTGGCAGCGAGCCTATGGTAATTGTGTGYCAGCMGCCGCGGTAA | EMP |
| 16S rRNA gene V4 region forward primer: R3D1 | AATGATACGGCGACCACCGAGATCTACACGCTGTGAATGTTCGATATGGTAATTGTGTGYCAGCMGCCGCGGTAA | EMP |
| 16S rRNA gene V4 region forward primer: R3D4 | AATGATACGGCGACCACCGAGATCTACACGCTTATGTTGACGGCTATGGTAATTGTGTGYCAGCMGCCGCGGTAA | EMP |
| 16S rRNA gene V4 region forward primer: R3D5 | AATGATACGGCGACCACCGAGATCTACACGCTAGTGTTTCGGACTATGGTAATTGTGTGYCAGCMGCCGCGGTAA | EMP |
| 16S rRNA gene V4 region forward primer: R3D8 | AATGATACGGCGACCACCGAGATCTACACGCTATTTCCGCTAATTATGGTAATTGTGTGYCAGCMGCCGCGGTAA | EMP |

^a^Barcodes for the 16S rRNA gene V4 region forward primers are indicated by the underlined sequences. Remaining sequence includes adapter, primer pad, linker, and actual primer sequence, which are all identical for the barcoded primers. Reactor samples are designated by replicate (R) and day of enrichment (D). An example is R1D4, a sample from the first reactor replicate, taken on the fourth day of enrichment.

^b^EMP = Earth Microbiome Project (Gilbert *et al*., 2014), ITS2 = internal transcribed spacer region 2

**Supporting Information References**

Weisburg WG, Barns SM, Pelletier DA, Lane DJ. 16S ribosomal DNA amplification for phylogenetic study. *J Bacteriol* 1991;173(2):697-703.

White TJ, Bruns T, Lee S, Taylor J. Amplification and direct sequencing of fungal ribosomal RNA genes for phylogenetics. In: Innis MA, Gelfand DH, Sninsky JJ, and White TJ (ed.). *PCR Protocols: A Guide to Methods and Applications*. San Diego, CA: Academic Press, 1990, 315-22.

Liu Y, Lai Q, Dong C, Sun F, Wang L, Li G, *et al*. Phylogenetic diversity of the Bacillus pumilus group and the marine ecotype revealed by multilocus sequence analysis. *PLoS ONE* 2013;8(11):e80097. doi: 10.1371/journal.pone.0080097

Gilbert JA, Jansson JK, Knight R. The Earth Microbiome project: successes and aspirations. *BMC Biol* 2014;12(1). doi:10.1186/s12915-014-0069-1
