# Supplementary material for: Identification of soil bacteria capable of utilizing a corn ethanol fermentation byproduct
Source: PLoS One. 2019 Mar 8;14(3):e0212685. doi: 10.1371/journal.pone.0212685 (PMC6407766; doi:10.1371/journal.pone.0212685)
Supplement: S2 Table — aCombinations performed are designated with “X” (DOCX) [file pone.0212685.s005.docx]

S2 Table. Binary growth combinations performed with laboratory strains and environmental isolates^a^

|  | M1 | M2 | M3 | M11 | UAE2 | UAE4 |
| --- | --- | --- | --- | --- | --- | --- |
| M2 | X |  |  |  |  |  |
| M3 | X | X |  |  |  |  |
| M5 | X | X | X |  |  |  |
| M6 | X | X | X |  |  |  |
| M7 | X | X | X |  |  |  |
| M11 | X | X | X |  |  |  |
| UAE2 | X | X | X | X |  |  |
| UAE4 | X | X | X | X | X |  |
| UAE5 | X | X | X | X | X | X |
| UAE10 | X | X | X | X |  |  |

^a^Combinations performed are designated with “X”
